# Ability of dog owners to identify their dogs by smell

**DOI:** 10.1038/s41598-021-02238-7

**Published:** 2021-11-23

**Authors:** Lucie Přibylová, Vendula Pilná, Ludvík Pinc, Hana Vostrá-Vydrová

**Affiliations:** Department of Ethology and Companion Animal Science, Faculty of Agrobiology, Food and Natural Resources, Czech University of Life Sciences Prague, Kamycka 129, 165 00 Prague 6, Czech Republic

**Keywords:** Physiology, Zoology

## Abstract

Several studies report that olfactory cues play an important role in human life; humans are essentially able to recognize other family members and friends by their odors. Moreover, recent studies report that humans are also able to identify odors of non-conspecifics. The aim of this study was to determine whether dog owners are able to identify their dogs by smell and distinguish the odor of their own dogs from those of other dogs. A total of 53 dog owners (40 females and 13 males of different ages) volunteered to take part in this study. A number of the participants (17) owned 2 dogs; these owners took part in the study twice (i.e., working with only one dog at a time). Sterile gauze pads were used to collect odor samples from the dogs. Each pad was placed in its own sterile glass jar (750 ml) with a twist off lid until the experiment commenced. Participants were asked to identify their own dog´s odor from a line-up of 6 glass containers. This experiment demonstrated that dog owners are capable of identifying their dogs by smell on a significant level. Results of this study additionally suggested that male owners outperformed their female counterparts in the identification process. Moreover, dog owners whose dogs were housed outside had a higher success rate in identification than did participants who kept their dogs indoors with them. The dog owners found it easier to identify dogs that had been neutered, fed dry dog food and bathed less frequently. In general, younger dog owners tended to have more success when attempting to identify their dogs than did their older counterparts.

## Introduction

Primates, including humans, have been considered a microsmatic species since at least the nineteenth century. This idea was based primarily on the size and shape of the olfactory bulb as well as on the fact that humans are not compelled to rely on olfaction to such a degree as are other mammals^1^. Later studies understandably focused on dogs to prove their olfactory superiority over humans^2–4^. However, recent results have suggested that human olfaction is not as feeble as previously thought, and when dealing with certain substances, humans were shown to perform as well as or even better than dogs and other mammals^5,6^. Further studies supported the idea of inferior human olfactory perception through the investigation of olfactory system morphology^7^, the repertoire of active genes that express olfactory receptor types^8,9^, and olfactory memory^10^; however, there is enough evidence to justify the opinion that human olfaction is not as poor as previously thought^5,11,12^. The terms microsmatic and macrosmatic are nevertheless justifiable when referring to species that possess or lack olfactory recesses^13^.

In the latter part of the twentieth century, a number of authors attempted to compare human olfactory sensitivity to that of other species. Because canine olfaction had always been considered exceptional, mostly dogs were chosen for testing and for comparative studies with humans^3,14–16^. Some studies in the 1950s and 1960s could not determine significant differences and suggested that human olfaction was on par with that of dogs^17,18^ in^15^. However, it is now an established fact that human olfaction is inferior to that of dogs^4,19^. Although human olfaction is considered one of the least important senses, it still plays a role in human life^20^. Odors are influential in social interaction settings^21^.They are used to perceive emotions^22,23^ and influence stress mechanisms^24^, and they also play an important role in the recognition of individuals^25–27^. Each individual has a distinct odor^28^, which can be influenced by many factors, such as the following: age^29^, diet^30,31^, hormonal changes^32^, and diseases or parasite infestation^33^.

Human olfaction is fully functional immediately after birth; newborns exhibit their preference for certain odors by attempting to get closer to their source^34^. According to Romantshik et al.^35^, newborns are essentially able to distinguish between known and unknown odors, and this ability enables them to locate the mother's nipple. Later on, children are capable of using their olfactory sense as effectively as humans in order to obtain information about food, their environment, or other humans^36^. Scientific studies have mostly focused on kin recognition. Studies have shown that mothers can recognize their children by smell several hours after giving birth, but the same studies indicate that fathers do not share in this ability^25,37^. Nevertheless, fathers are able to identify scents of their children once they reach a certain age (8–9 years). A father’s ability to recognize his child through olfaction may be influenced by the nature of the child’s upbringing. Fathers who can recognize their children more easily have a friendlier approach towards child-rearing^38^. These abilities are not limited to kin recognition; it is generally believed that women can memorize the odor from newborn children of other mothers. This was demonstrated in one study by Kaitz and Eidelman^26^, who tested childless women for their ability to recognize infants through smell. Most of the participants correctly identified an infant after having held it in their arms for one hour. In another study by Roberts et al.^39^, human participants were able to distinguish between the odors of twins at the rates better than those of mere chance. As described above, humans can recognize other humans whether they are kin or not; moreover, they are essentially able to categorize body odors according to age^29^or sex^22^.

This evidence suggests that humans can identify other conspecifics and obtain certain information about them^22,29^.

Nevertheless, to our knowledge, only a handful of studies have demonstrated the ability of humans to identify individual odors of other mammals,such as: mice^40^ and older gorillas,^41^ and only one known study involving dogs was carried out^42^. In contrast to these findings, Courtney and Wells^43^ could not confirm the ability to identify individual cat odors; only 52% of the twenty-five participants correctly identified their cats. The authors attribute the low success rate to the fact that cats emanate milder odors than do dogs due to more frequent and intense self-grooming habits.

All of the studies cited above differ in their odor sampling methodology: Wells and Hepper^42^ used a collection blanket that was placed in the dog’s bed for three consecutive nights; Hepper and Wells^41^ placed a cotton towel in the gorilla’s sleeping area from late afternoon until the next morning; and Courtney and Wells^43^ rubbed a blanket over the cats back. Wells and Hepper^42^ based odor identification on a comparison of only two samples. This led to a high probability of successful recognition due to not only the low number of samples but also due to the fact that correct identification could be based solely on the recognition of the novel odor^41^. A higher number of targets is therefore required in order to eliminate random successful recognition and reliably demonstrate the ability of a human to successfully identify his/her own dog by smell.

To explore this topic further, we asked dog owners to identify their dogs odor from six samples. We hypothesized that the owners would be able to correctly identify their dog odors and that several other factors could have influenced success rates.

## Results

A total of 40 female dog owners and 13 male dog owners aged between 3 and 72 years participated in this study. Several participants (17) had two dogs; these owners took part in the study twice (i.e., working with only one dog at a time). The mean and median ages were 32 and 29 years respectively.

**Figure 1:**
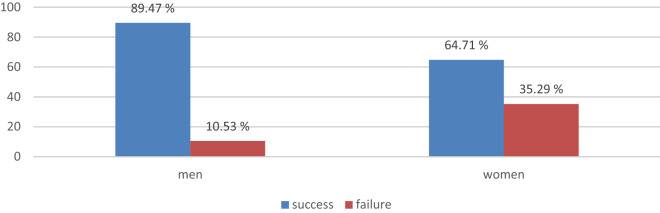
Success rate of correct identification in men and women.

The results of this study indicate that the owners were able to distinguish their dogs from the other five dogs at a significance level of *P* < 0.05. In total, 71.43% of the participants correctly identified their dogs (28.57% did not). We found significant differences between men and women (χ^2^_(1)_ = 4.16; *P*-value = 0.04); men had a higher success rate (89.47%; N = 17 men) than did their female counterparts (64.71%; N = 33 female) (Fig. 1).

Table 1 illustrates the estimated statistical significance of some fixed effects included in the model. Based on a *P*-value of *P* < 0.05, we revealed statistically significant differences in some variables. Owners who kept their dogs outside correctly identified their odors at a significantly higher rate (*P* = 0.0394) than did owners who housed their dogs indoors. Participants who fed their dog dry dog food also had a significantly higher rate of success (*P* = 0.0270) than did owners who fed their dogs raw meat. The frequency at which the dogs were bathed had a significant effect (*P* = 0.0314) on the owners’ ability to identify their dogs. Owners who bathed their dogs frequently were less likely to correctly identify their scent.

**Table1:** Estimated parameters for each variable.

Variable	Code (Number of dogs)	Estimation	SE	Probability (%)	SE (%)	*P*-value
Neutered	No (55)	1.2771	0.5878	78.20	10.02	0.0534
	Yes (15)	3.9306	1.3692	98.07	2.59	
Housing	Outdoor (15)	3.8944	1.2419	98.01	2.43	0.0394
	Indoor (55)	1.3132	0.7223	78.81	12.06	
Diet	Dry dog food (50)	3.5102	0.8935	97.10	2.52	0.0270
	Raw meat (BARF) (20)	1.6975	0.9129	84.52	11.94	
Bath	–	− 0.1670	0.0759	–	–	0.0314
Age of the Owner	–	− 0.0468	0.0232	–	–	0.0477

Finally, the younger the dog owners the higher the success rate (estimation = − 0.0468), (*P* = 0.0477). Differences in rates of successful identification between neutered and unneutered dogs were not statistically significant; however, the *P*-value (*P* = 0.0534) showed a tendency towards statistical significance. For more details, see Table 1.

All correlations between variables (sex of the owner, castration, diet, housing) were statistically significant (*P* < 0.05); they were, however, shown to be weak (0.07–0.33). Causality between analyzed factors was not proven. For more details, see Table 2.

**Table 2:** Correlation factors between variables (Fisher's Exact Test).

	Housing	Neutered	Owner sex
Diet	0.232	0.330	0.183
Housing		0.296	0.254
Neutered			0.073

## Discussion

The main aim of this work was to determine whether dog owners are able to distinguish their own dogs from others by smell. To do so, we asked for volunteers to take part in a double-blind study. Each participant was asked to identify his dog odor from a line-up of 6 odors kept in separate glass containers, where was one target sample of own dog odor and five non-target samples. Non-target samples were randomly collected from other dogs.

Results of this study actually showed that humans are able to distinguish their own dogs from others by smell. Several factors seem to play an important role in successful identification. Dogs that are kept outdoors, bathed less frequently, and fed dry dog food were more likely to be correctly identified by their owners. Moreover, younger male participants enjoyed higher rates of success than did their older female counterparts. Results from the present study correspond to those of a previous study by Wells and Hepper^42^. In contrast to the Wells and Hepper^42^ study, in which participants were asked to distinguish between just two odors, our study required participants to identify their dogs from an assortment of six dog odors. This strategy gave us a good indication that the episodes of correct identification could not have all occurred by chance.

In contrast to many studies that report greater olfactory capabilities in females^26,44,45^ this study indicates an opposite trend since the male participants had higher rates of success in identifying their dogs. These results are somewhat surprising. As Brand and Millot^46^ mentioned in their review paper, there is a common belief that women possess a greater olfactory sense than men. Moreover, it is very difficult to draw definitive conclusions on this topic since different studies utilize various cues and methodologies. Increased identification skills based on olfactory sense were also later reported by Olsson et al.^11^, who examined whether males or females possess a greater olfactory ability to identify a platonic friend. Although data from that study are not deemed statistically significant due to the low sample size, female participants were reported to have higher rates of success. Based on data from recent separate meta-analyses carried out by Sorokowski et al.^45^, women generally outperform men with respect to all aspects of olfactory abilities (identification, discrimination, and threshold).

The previously mentioned study by Wells and Hepper^42^, which examined the ability of humans to distinguish their dogs from others by smell, reported that men enjoyed rates of success similar to those of women. It is rather complicated to determine whether females or males make better sniffers. Moreover, it is important to mention that the results of our study could have been influenced by the high number of female participants.

To our knowledge, no study has yet to investigate whether it is easier to identify by smell a dog that is housed outdoors or one that resides indoors as part of a household. Nevertheless, results of this study indicate that participants were more likely to identify dogs housed outdoors than their indoor counterparts. This could be explained by the fact that prolonged exposure to odor leads to sensory adaptation, which is common in olfaction^47^. Contrarily, Cain et al.^44^ reported that the longer an individual is exposed to an odor the easier it is to identify said odor. Moreover, dogs that are housed indoors also emanate odors acquired from the household or owner, thereby making it easier to identify their scents^48^.

Another variable that was included in this study was the diet of the dog. Results of this study suggest that dog nutrition had a statistically significant effect on olfactory identification. Dogs fed dry food were more easily identified by their owners than those fed raw meat (B.A.R.F). One reason for this, although unlikely at first glance, could be that owners prefer the smell of dogs that are not fed raw meat. As Havlicek and Lenochova^31^ reported in their study, women preferred the scent of men who eat a plant-based diet to that of men who consume large amounts of meat. Those results were later confirmed by Zuniga et al.^30^, who determined that women consider the scent of a man more pleasant when his diet is richer in fruits and vegetables. This leads us to believe that since most of the participants in this study were female, it is possible that they found the odor of their own dogs, which were fed dry food, more pleasant, thereby making it easier for them to distinguish their own dogs from others.

The last variable tested in this study was owner age. A larger sample size would have been needed in order to acquire more accurate results for this particular variable. This variable was calculated as a regression. Our results indicated that the older the owner was, the less likely they were to recognize their dog. Regardless of the low sample size, results of this study are in agreement with those of previous studies, which have reported decreases in olfaction ability with increased age^49,50^.

The correlations between variables turned out to be weak (between 0.07 and 0.33). As mentioned before, the results could be slightly biased due to the low number of participants when divided into the four categories. Nevertheless, this study is only the second of its kind to deal with this topic, and its aim was to determine whether humans are able to identify their own dogs by their odors, thereby confirming the results of a small study from Wells and Hepper^42^. Additionally, it is important to note that most of the participants are not the sole owner of their dogs. Therefore, some of the correlations may be misleading since an additional owner (not a study participant) might have influence over certain variables (housing, diet) presented in the study.

In conclusion, the main finding of this study was that dog owners are able to identify their own dogs by smell at a significant level. In addition, the type of dog housing, dog diet, bathing frequency of the pets, and owner age did influence the owners’ ability to distinguish their dogs from others. These results clearly show that human olfactory sense is not as poor as previously thought, and that humans are able to identify not only their conspecifics but also other mammals. In future studies, it would be very useful to have better control over all variables in order to study their influence in greater depth.

## Material and methods

### Participants

A total of 40 female dog owners and 13 male dog owners aged between 3 and 72 years participated in this study. Several participants (17) had two dogs; these owners took part in the study twice (i.e., working with only one dog at a time). The mean and median ages were 32 and 29 years respectively. Participants were recruited either from the Czech University of Life Sciences in Prague or obedience schools.

All of the animal procedures were approved by the Expert Commission for the Welfare of Experimental Animals of the Czech University of Life Sciences (Permit No.: 63479/2016-MZE-17214) and conducted in accordance with guidelines that were established by European Council Directive 2010/63/EU and accordance with the ARRIVE guidelines^51^.

All Participants had been informed in advance about the course of the experiment and signed a written consent to participate in the study, which took place at the Canine Behaviour Research Center of the Czech University of Life Sciences, Prague. All children who participated in this study did so in the presence of their parents and with their full written consent. Subjects took part in the study anonymously; therefore personal data were not collected. All of the human procedures were performed in accordance with the Declaration of Helsinki and have been approved by licenses Ethics Committee (Ethics Committee of Czech University of Life Sciences Prague).

### Odor collection

Samples were collected between October 2015 and February 2017.

Sterile gauze pads were used to collect dog odor samples, which were placed in sterile glass jars with twist-off lids immediately after collection. This sampling method is normally used for criminal investigation in the Czech Republic^52^ and has been used in other studies^53,54^. The sterile gauze pads are made of a combination of viscose and polyester and are primarily used for medical purposes. During sampling, these pads were covered with aluminum foil to prevent contamination.

New material was used for each odor sampling. Prior to the experiment, the glass jars and their lids were washed with detergent for 15 min in an ultrasonic cleaner at 70 °C. They were subsequently sterilized at 180 °C for 35 min.

In order to reduce possible contamination with other household odors, the samples were collected in an outdoor environment. Sampling took place only during acceptable weather conditions, which meant without rain and/or strong wind.

Owners were asked to take odor samples from their own dogs and were shown proper sampling procedures. The owners collected the samples in the presence of the experimenters.

Prior to sample collection, dog owners were required to wash and dry their hands thoroughly. A sterile gauze pad was placed under the dog's collar, where it remained for one hour. It was then placed into a sterilized glass container with a lid and labelled with number code. The number codes were recorded and the gauze pads were sealed until the end of the experiment. The samples were checked for the presence of any dog hair, which might have led to the animal being identified visually rather than through olfaction.

During sampling, the dogs were closely monitored to ensure that they did not lose their sterile gauze pads or contaminate them with other odors. Dog owners were instructed not to wash their dog or use any chemical products on their coats for at least two weeks prior to the experiment.

### Experiment

Six glass jars were randomly placed in a lineup in front of each human participant. This was a double-blind experiment, meaning neither the dog owners nor the experimenters were aware of which jar contained the correct sample. Further identification was possible only according to the (four) number codes. Each collection of jars comprised one target sample and five non-target samples. Non-target samples were randomly collected from other dogs.

Participants were allowed to sniff for as long as they desired before telling the experimenter the number code of the jar which they believed contained the target sample. The experimenter stayed in a separate room for the duration of the identification portion of the experiment in order not to influence the results.

The experiment took place in a room that had been ventilated for 15 min prior to the start of the experiment. Only the experimenter and the participant were present in the room.

### Statistical analyses

Exploratory data analysis was used for basic data description.

To compare the independence of two variables in a contingency table, the Pearson Chi-square (Fishers exact factor) test was used.

Calculations were performed using a GLIMIX procedure that fit a logistic linear model used in our study (calculated with SAS Inst. Inc., Cary, NC). The dependent variable y_i_ can take the value 1 (participant has correctly identified his/her own dog) with a probability of π_i_ . The dependent variable y_i_ can take the value 0 (participant did not correctly identify his/her own dog) with a probability of 1- π_i_ (observation *i)*. We used the following model to determine probability of correct identification:

$$log\left( {\frac{{\pi_{ijkl} }}{{1 - \pi_{ijkl} }}} \right) = Neutered_{i} + Housing_{j} + Diet_{k} + b_{1} Bath + b_{2} OwnerAge$$

*Neutered*_*i*_ was a fixed effect of the ith Neutered (*i* = no or yes), *Housing*_*j*_ is a fixed effect of the jth Housing (j = indoor or outdoor), *Diet*_*k*_ is a fixed effect of the kth Diet (k = dry dog food or meat), *Bath*_*l*_ is a fixed effect of the lth Bath (l = bathing frequency), *b1 is* the regression coefficient for bathing frequency and $b_{2}$ *is* the regression coefficient for owner age.

The model (with selected variables) showed values of Akaike information criterion (AIC = 66.31).

### Description of the data

Odors from 35 male dogs and 35 bitches were collected. Of these 70 dogs, 15 were neutered at the time of the experiment. The dogs involved in this study were of various breeds and sizes: large-sized breeds (62.9%); small-sized breeds (15.7%); medium-sized breeds (14.3%); and mixed-breeds (7.1%). The dogs ranged from 5 months to 16 years of age.

## Supplementary Information

Supplementary Information.
