# Supplementary material for: Ability of dog owners to identify their dogs by smell
Source: Sci Rep. 2021 Nov 23;11:22784. doi: 10.1038/s41598-021-02238-7 (PMC8610967; doi:10.1038/s41598-021-02238-7)
Supplement: Supplementary file 1 — Supplementary Information. [file 41598_2021_2238_MOESM1_ESM.pdf]

Table of all data:

Dog	Success	Sex of the owner	Owners age (years)	Castrated	Housing	Bath	Diet
1	+	F	29	-	Indoor	2	Dry food
2	+	F	29	-	Outdoor	2	Dry food
3	-	F	23	-	Indoor	12	Meat
4	+	F	24	-	Indoor	6	Dry food
5	+	M	25	-	Indoor	6	Dry food
6	+	F	25	-	Outdoor	2	Dry food
7	+	F	25	-	Outdoor	2	Dry food
8	+	F	30	-	Indoor	3	Dry food
9	-	F	58	-	Indoor	3	Dry food
10	+	F	53	-	Outdoor	0	Meat
11	-	F	53	-	Outdoor	0	Meat
12	-	F	44	-	Indoor	2	Meat
13	+	F	48	Castrated	Indoor	12	Dry food
14	+	F	43	-	Indoor	3	Meat
15	-	F	43	-	Indoor	3	Meat
16	+	F	43	-	Outdoor	0	Dry food
17	+	M	46	-	Outdoor	0	Dry food
18	+	F	16	-	Outdoor	0	Dry food
19	+	F	26	-	Indoor	0	Dry food
20	+	F	26	Castrated	Indoor	5	Dry food
21	+	M	32	Castrated	Indoor	5	Dry food
22	+	M	15	-	Outdoor	0	Meat
23	+	M	15	-	Outdoor	0	Meat

24	-	F	40	-	Indoor	4	Dry food
25	+	M	45	-	Outdoor	0	Dry food
26	+	F	37	-	Outdoor	0	Dry food
27	+	F	25	Castrated	Indoor	2	Dry food
28	+	M	25	Castrated	Indoor	2	Dry food
29	+	F	26	-	Indoor	4	Dry food
30	+	F	52	-	Outdoor	0	Dry food
31	-	F	27	-	Indoor	4	Meat
32	-	F	41	-	Indoor	3	Dry food
33	+	F	41	Castrated	Indoor	3	Dry food
34	+	F	8	-	Indoor	3	Dry food
35	+	F	8	Castrated	Indoor	3	Dry food
36	+	F	5	-	Indoor	3	Dry food
37	+	F	5	Castrated	Indoor	3	Dry food
38	-	F	28	-	Indoor	12	Dry food
39	+	M	33	-	Indoor	12	Dry food
40	-	F	63	-	Indoor	8	Dry food
41	+	F	63	-	Indoor	8	Dry food
42	+	F	26	-	Indoor	3	Dry food
43	+	F	27	Castrated	Indoor	1	Dry food
44	+	F	21	-	Indoor	0	Meat
45	+	F	50	-	Indoor	0	Dry food
46	+	F	41	-	Indoor	4	Meat
47	-	F	41	-	Indoor	4	Meat
48	+	M	38	-	Indoor	4	Meat
49	-	M	38	-	Indoor	4	Meat

50	-	F	27	Castrated	Indoor	12	Dry food
51	-	F	27	-	Indoor	24	Dry food
52	-	M	32	-	Indoor	12	Dry food
53	+	M	32	Castrated	Indoor	24	Dry food
54	-	F	38	-	Indoor	2	Meat
55	-	F	4	-	Indoor	2	Meat
56	-	F	7	-	Indoor	12	Dry food
57	-	F	72	-	Indoor	12	Dry food
58	-	F	48	-	Indoor	0	Dry food
59	+	F	39	-	Indoor	4	Dry food
60	+	F	40	-	Indoor	0	Dry food
61	+	F	26	Castrated	Indoor	12	Dry food
62	+	F	26	Castrated	Indoor	6	Dry food
63	+	M	28	Castrated	Indoor	12	Dry food
64	+	M	28	Castrated	Indoor	6	Dry food
65	+	F	3	-	Indoor	12	Dry food
66	+	M	6	-	Indoor	12	Dry food
67	+	M	42	-	Outdoor	0	Meat
68	+	M	42	-	Outdoor	0	Meat
69	+	M	26	-	Outdoor	0	Meat
70	+	M	26	-	Outdoor	0	Meat
